# Supplementary material for: Attention module improves both performance and interpretability of four‐dimensional functional magnetic resonance imaging decoding neural network
Source: Hum Brain Mapp. 2022 Feb 25;43(8):2683–92. doi: 10.1002/hbm.25813 (PMC9057093; doi:10.1002/hbm.25813)
Supplement: Supplementary file 1 — Figure S1 Interaction between features and attention masks at low‐level. An example showing the feature maps before and after masking of a randomly selected subject. (a) and (b) Examples of Stage 1. (c) and (d) Examples of Stage 2. The focused layouts of the attention mask were similar across different tasks, which tended to highlight the useful areas while diminishing the noise areas. Figure S2 Interaction between features and attention masks at high‐level. An example showing the feature maps before and after masking of a randomly selected subject. (a) and (b) Examples of Stage 3. (c) and (d) Examples of Stage 4. The focused layouts of the attention masks varied across different tasks and were remarkably task‐specific. Figure S3 Contrast images across task conditions. (a) The attention mask of Figure 3b, averaged across the seven tasks. (b)–(h) The contrasts between the attention mask of each task and the average mask. The highlighted areas varied across tasks and were around the brain areas related to each task, for example, temporal lobes in the language (story) task, left S1 and M1 in the motor (right hand) task, and the temporal–parietal junction in the social (mental) task. [file HBM-43-2683-s001.docx]

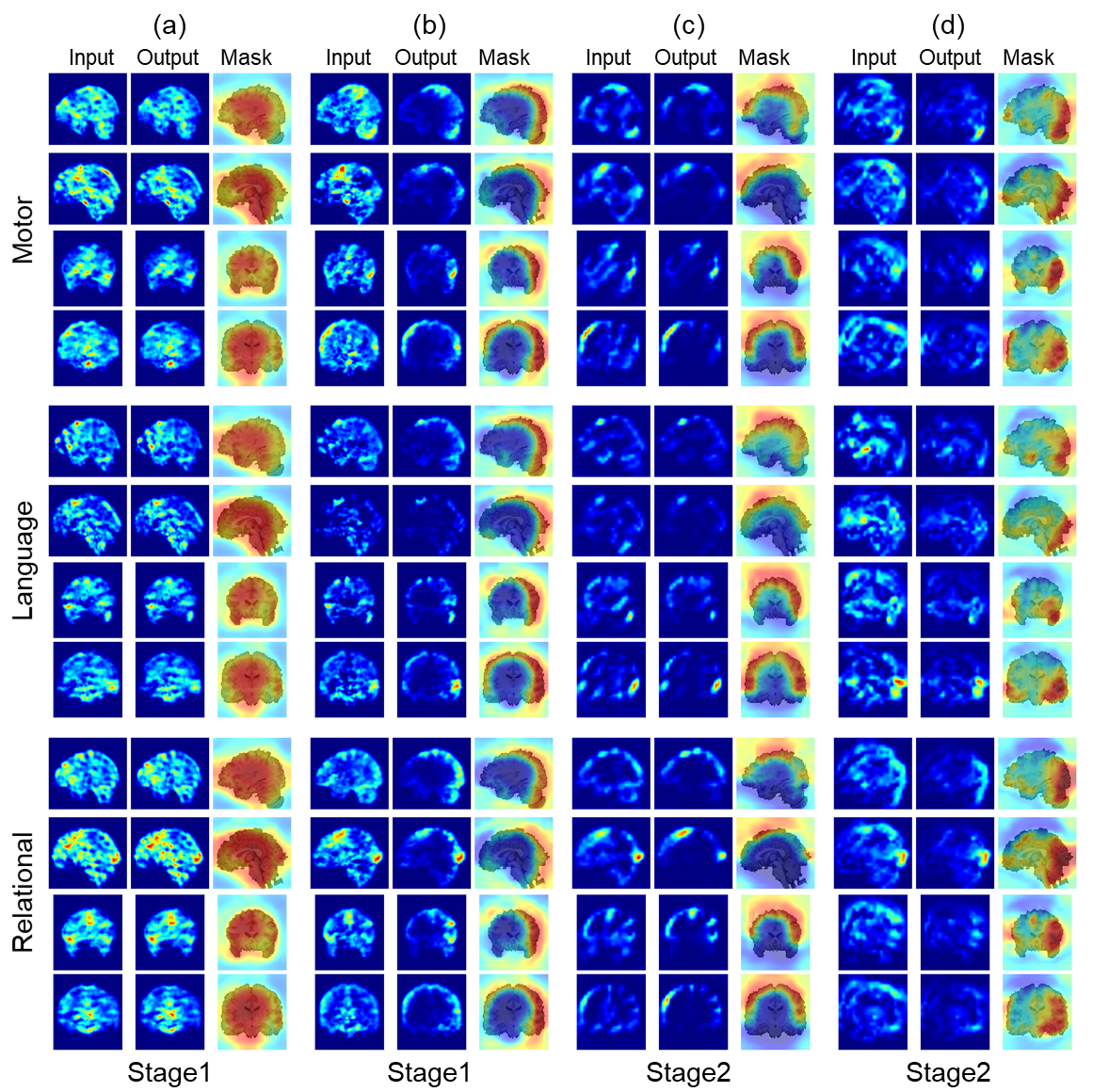


**Figure S1 Interaction between features and attention masks at low-level.**

An example showing the feature maps before and after masking of a randomly selected subject. (a)-(b) Examples of stage1. (c)-(d) Examples of stage2. The focused layouts of the attention mask were similar across different tasks, which tended to highlight the useful areas while diminishing the noise areas.

**
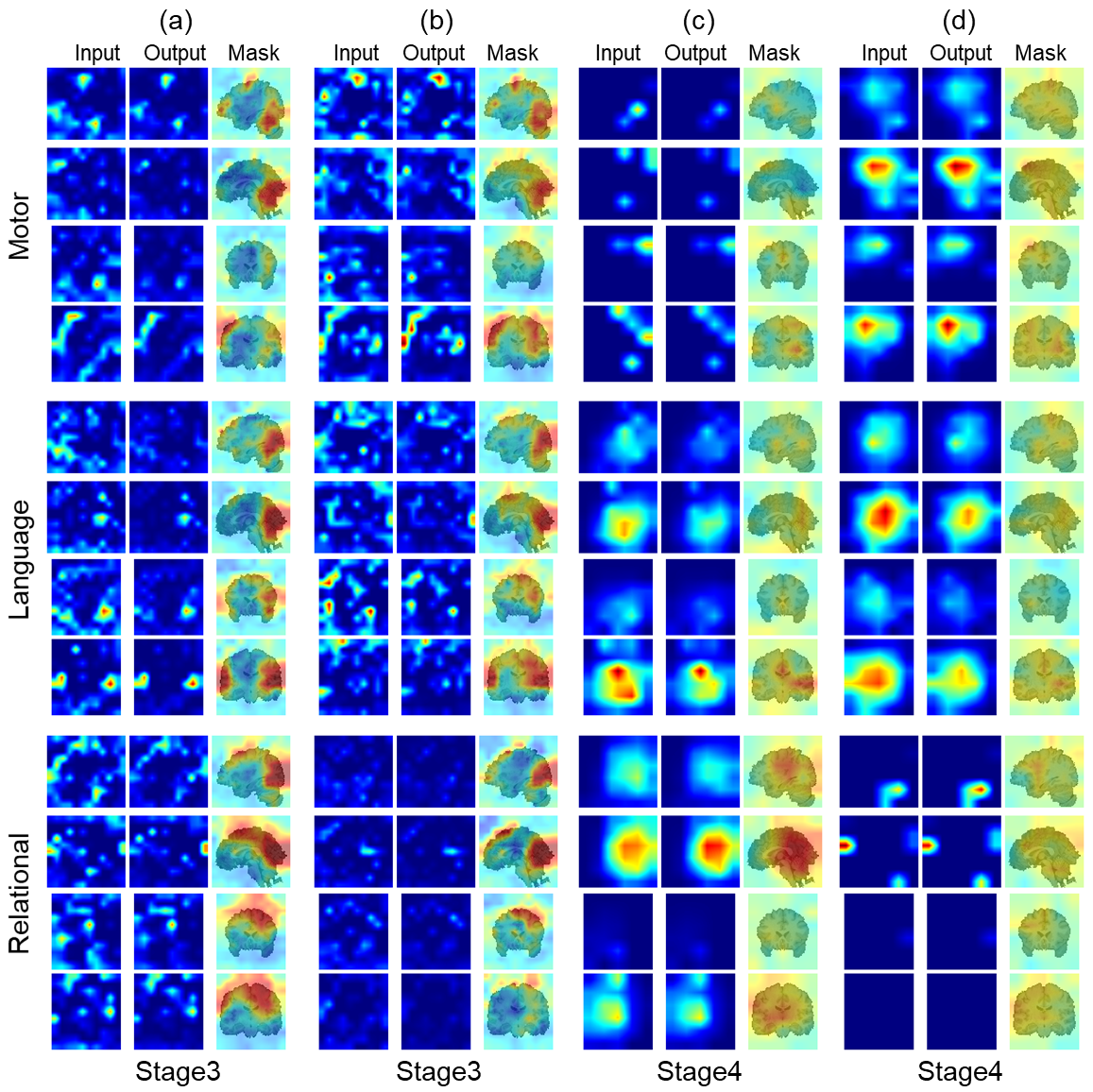
**

**Figure S2 Interaction between features and attention masks at high-level.**

An example showing the feature maps before and after masking of a randomly selected subject. (a)-(b) Examples of stage3. (c)-(d) Examples of stage4. The focused layouts of the attention masks varied across different tasks and were remarkably task-specific.

**
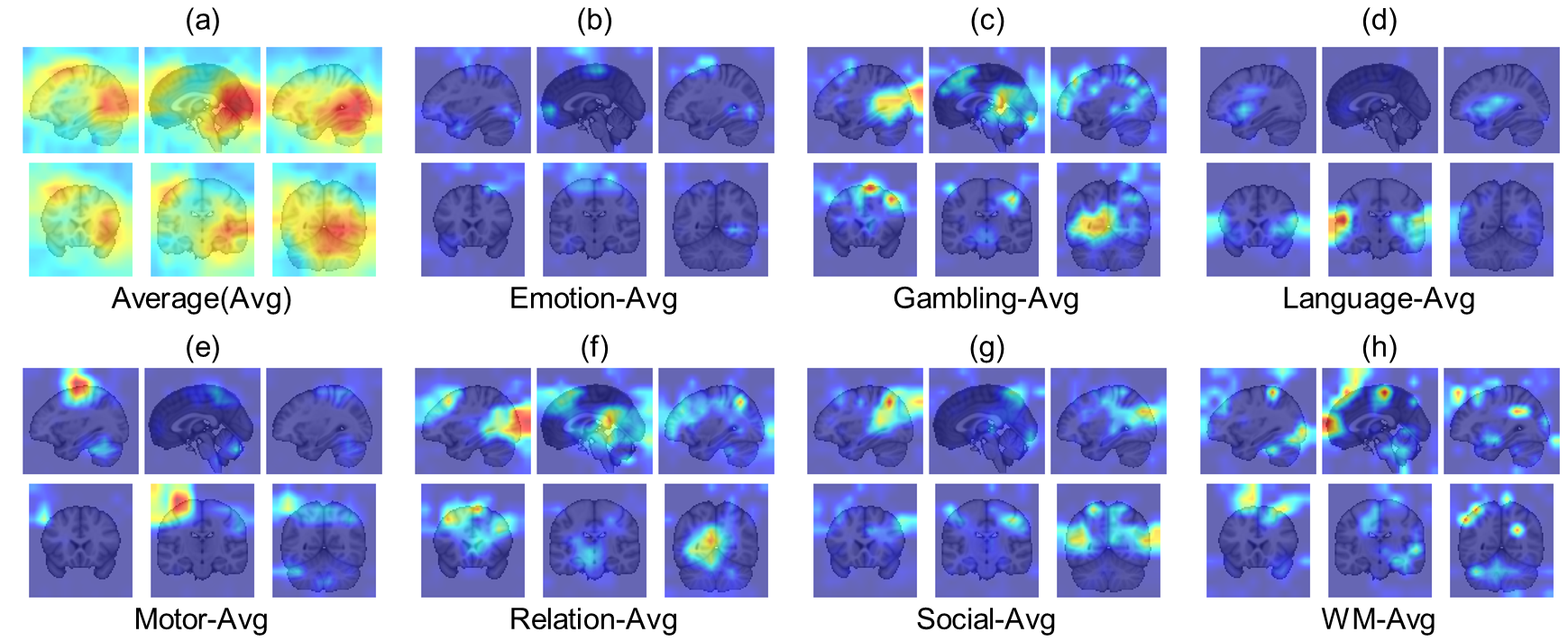
**

**Figure S3 Contrast images across task conditions.**

(a) The attention mask of Figure 3b, averaged across the 7 tasks. (b)-(h) The contrasts between the attention mask of each task and the average mask. The highlighted areas varied across tasks and were around the brain areas related to each task, e.g. temporal lobes in the Language (story) task, left S1 & M1 in the Motor (right hand) task, and the temporal-parietal junction in the Social (mental) task.
